# Baculovirus entry into the central nervous system of *Spodoptera exigua* caterpillars is independent of the viral protein tyrosine phosphatase

**DOI:** 10.1098/rsob.230278

**Published:** 2024-02-21

**Authors:** Simone N. Gasque, Yue Han, Iris van der Ham, Dorothy van Leeuwen, Monique M. van Oers, Alexander Haverkamp, Vera I. D. Ros

**Affiliations:** ^1^ Laboratory of Virology, Wageningen University and Research, Droevendaalsesteeg 1, 6708 PB Wageningen, The Netherlands; ^2^ Laboratory of Entomology, Wageningen University and Research, Droevendaalsesteeg 1, 6708 PB Wageningen, The Netherlands

**Keywords:** Autographa californica multiple nucleopolyhedrovirus, *Spodoptera exigua*, parasite-induced behavioural manipulation, central nervous system, protein tyrosine phosphatase, neuroparasitology

## Abstract

Neuroparasitism concerns the hostile take-over of a host's nervous system by a foreign invader, in order to alter the behaviour of the host in favour of the parasite. One of the most remarkable cases of parasite-induced host behavioural manipulation comprises the changes baculoviruses induce in their caterpillar hosts. Baculoviruses may manipulate caterpillar behaviour in two ways: hyperactivity (increased movement in the horizontal plane) and/or tree-top disease (movement to elevated levels in the vertical plane). Those behavioural changes are followed by liquefaction and death of the caterpillar. In Autographa californica multiple nucleopolyhedrovirus (AcMNPV)-infected *Spodoptera exigua* caterpillars, an enzymatic active form of the virally encoded protein tyrosine phosphatase (PTP) is needed for the expression of hyperactivity from 3 days post infection (dpi). Using eGFP-expressing recombinant AcMNPV strains, we show that infection of the caterpillar's central nervous system (CNS) can be observed primarily from 3 dpi onwards. In addition, we demonstrate that the structural and enzymatic function of PTP does not play a role in infection of the CNS. Instead we show that the virus entered the CNS via the trachea, progressing caudally to frontally through the CNS and that the infection progressed from the outermost cell layers towards the inner cell layers of the CNS, in a PTP independent manner. These findings help to further understand parasitic manipulation and the mechanisms by which neuroparasites infect the host nervous system to manipulate host behaviour.

## Graphical abstract

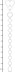

## 1. Introduction

Many parasites and pathogens modify the behaviour of their host to increase transmission [1–10]. A textbook example of behavioural alteration is the one expressed by baculovirus-infected caterpillars (lepidopteran larvae) [11,12]. Baculoviruses have a circular double-stranded genome (81–178 kilo base pairs) that is encompassed in enveloped, rod-shaped nucleocapsids [13,14]. Nucleocapsids are packaged into two types of virions; budded virions (BVs) which are responsible for cell to cell infections and occlusion-derived virions (ODVs) enclosed in occlusion bodies (OBs) which are needed for host to host infection [15,16]. Baculoviruses trigger two forms of parasite-induced behavioural alteration in caterpillars: hyperactivity and tree-top disease [12,17–20]. The two behavioural alterations are expressed after the oral infection of caterpillars through virus-contaminated vegetation that the caterpillars feed on, and are observed as an increased movement either in the horizontal plane (hyperactivity) or in the vertical plane (tree-top disease). Soon after, the caterpillars liquefy and die. Tree-top disease is argued to be one of the first known cases of parasite-induced behavioural alteration, as it was already reported in 1891 [18].

Although the underlying mechanisms behind behavioural inductions due to parasite/pathogen infections have scarcely been elucidated [21], it was within the field of baculoviruses that a parasite gene responsible for an expressed host behaviour was identified for the first time [22]. Viral protein tyrosine phosphatase (*ptp*) was the factor behind the offset of hyperactivity from 3.75 to 4.75 days post infection (dpi) for Bombyx mori nucleopolyhedrovirus (BmNPV)-infected *Bombyx mori* neonate caterpillars [22]. Since then *ptp* has been shown to be involved in the expression of hyperactivity in a few other baculovirus-caterpillar systems [12], including Autographa californica multiple nucleopolyhedrovirus (AcMNPV) in caterpillars of the beet armyworm *Spodoptera exigua* [23]. More specifically, the gene encoding PTP or homologues thereof are found within alphabaculoviruses in the clade termed group I NPVs, and PTP-induced hyperactivity seems a conserved mechanism for this clade [23,24]. PTPs form a large family of enzymes defined by the active-site signature motif HCX_5_R, where the nucleophile cysteine residue is essential for the catalysis [25–27]. PTPs from baculoviruses belong to the dual-specific PTPs (DUSPs), which can dephosphorylyse phosphotyrosine (pTyr), as well as phosphoserine (pSer) and phosphothreonine (pThr) residues, in contrast to the classical PTPs where pTyr is the only substrate [26–28]. Furthermore, PTP encoded by AcMNPV is even more effective as an RNA 5′-triphosphatase (RNA capping enzyme), which classifies it as part of the dual specificity protein phosphatase 11 (DUSP11) subfamily, similar in function to the human PIR1 [25,27,29–32] (NCBI reference domain: cd17665).

Within baculoviruses, two *ptp* genes are known, *ptp* and *ptp2*, that are phylogenetically unrelated and have low protein similarity, but belong to the same PTP protein family [23]. While *ptp* is important in behavioural manipulation and is present in all group I NPVs, *ptp2* plays a role in the induction of apoptosis and is present in some but not all baculoviruses [33]. The baculovirus AcMNPV (belonging to group I NPVs of the alphabaculoviruses) encodes for only one PTP [23,30]. The baculoviral *ptp* gene very likely derived from the genome of an ancestral lepidopteran host, and was presumably only incorporated in an ancestral baculoviral genome once, wherefrom it afterwards spread within group I NPVs [23].

The role of PTP in the expression of hyperactivity in caterpillars seems to be dependent on the virus–host combination. *Bombyx mori* fifth instar caterpillars infected with a mutant BmNPV, which expressed solely the structural properties and not the enzymatic function of PTP, displayed hyperactivity [34]. In AcMNPV-infected third instar *S. exigua* caterpillars, in contrast, the enzymatic activity was pivotal for the display of hyperactivity at 3 dpi [23]. Furthermore, based on the reduced viral propagation in brain tissues after *ptp*-deletion from the BmNPV genome, Katsuma *et al*. [34] hypothesized that PTP aids in the infection of brain tissues, which would have a direct influence on the expression of hyperactivity. As previous studies have shown the presence of baculoviruses in the host CNS [34–37], the modulation of the CNS by baculoviruses forms a needed target of research within this case of baculoviral-induced behavioural changes and more generally in the field of neuroparasitology [38].

**Figure 1.**
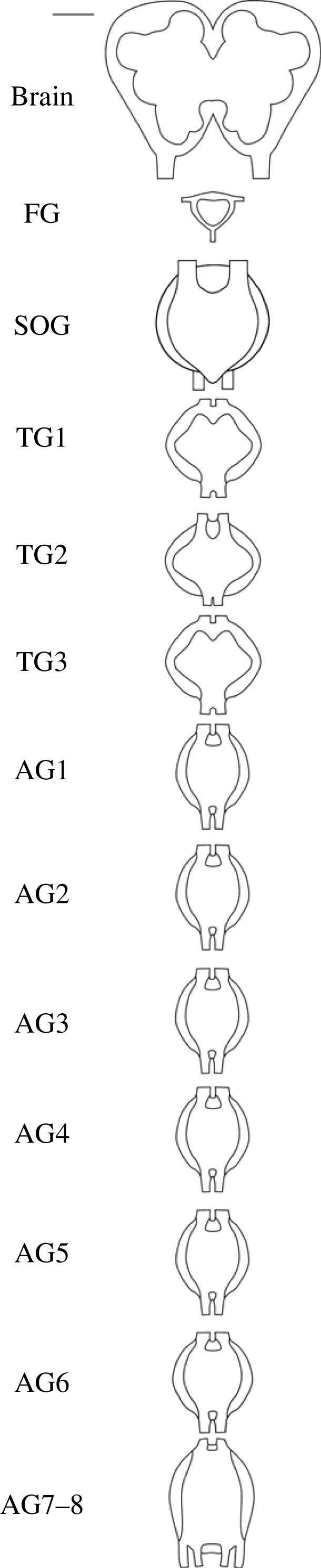
Schematic overview of the central nervous system of a mid-third instar *S. exigua* caterpillar, consisting of the supraoesophagal ganglion (brain), followed by the suboesophagal ganglion (SOG), three thoracic ganglia (TG1 to TG3) and seven abdominal ganglia (AG1 to AG7–8; abdominal ganglia 7 and 8 are fused). Each ganglion is connected to the adjacent ganglion by two connectives (not fully depicted). The much smaller frontal ganglion (FG) is connected to the brain which it is placed acutely caudally to. Scale bar (top left) represents 50 µm.

For caterpillars, the CNS is organized in different ganglia. Frontally to caudally the CNS starts with the supraoesophagal ganglion (brain) connected to both the much smaller frontal ganglion (which is placed acutely caudally to the brain) and the suboesophagal ganglion (SOG), the latter by the circumoesophagal connectives. The SOG is followed by the first to third thoracic ganglia, and subsequently seven abdominal ganglia [39] (figure 1).

Here, we test whether wild-type (WT) AcMNPV can infect the CNS of *S. exigua*, and when and where the virus is visible in the ganglia, and whether it is present on a superficial or internal level. Additionally, we investigate the route of infection by analysing the different ganglia during the course of infection. Moreover, we test whether PTP plays a role in CNS entry by comparing infections by WT AcMNPV and mutant AcMNPV strains either lacking *ptp*, containing a *ptp* gene with an inactivated catalytic site, or where the *ptp* gene deletion has been repaired, respectively. Lastly, we analyse whether any of these strains shows detectable differences in infection patterns for the different ganglia of the CNS. We found that AcMNPV seems to infect the CNS through the trachea, and based on the infection levels over time of the different ganglia, the infection progress caudally to frontally. Furthermore, all recombinant viruses tested here could infect *S. exigua* and the CNS, providing further insights into the mechanisms by which baculoviruses can highjack the host's nervous system and express their extended phenotype.

## 2. Material and methods

### 2.1. Insect larvae and cells

*Spodoptera exigua* larvae (originally derived from Andermatt Biocontrol, Switzerland, in 2014) were reared under a 14 h : 10 h light : dark photoperiod at 25.5°C and 50% relative humidity on artificial diet (primarily consisting of polenta, wheat germ and yeast) [40]. Disposable plastic trays covered with paper tissue and a lid were used as rearing containers for groups of 50 (smaller instars) to maximum 35 (larger instars) larvae. Late fifth instars were transferred to a plastic tray containing vermiculite to facilitate pupation. Pupae were collected and transferred to cylindrical containers lined with paper sheets for egg deposition, with around 70 pupae per container. Adult moths were provided with water. Collected eggs were surface sterilized using 10% formaldehyde vapour and subsequently put at 28°C until hatching, after which larvae were transferred to 25.5°C.

The *Spodoptera frugiperda* derived cell line Sf9 was cultured as a monolayer in SF900II medium supplemented with 5% fetal bovine serum (FBS) and 50 µg ml^−1^ gentamycin (all from Invitrogen) for the subsequent transfection with all recombinant bacmids (§2.3), except for the AcMNPV Δ*ptp* bacmid (see below), which was transfected into cells cultured in Gibco Sf-900 II serum free medium (Life Technologies, Fisher Inv.) and 50 µg ml^−1^ gentamycin.

### 2.2. Generation of recombinant bacmids

**Figure 2.**
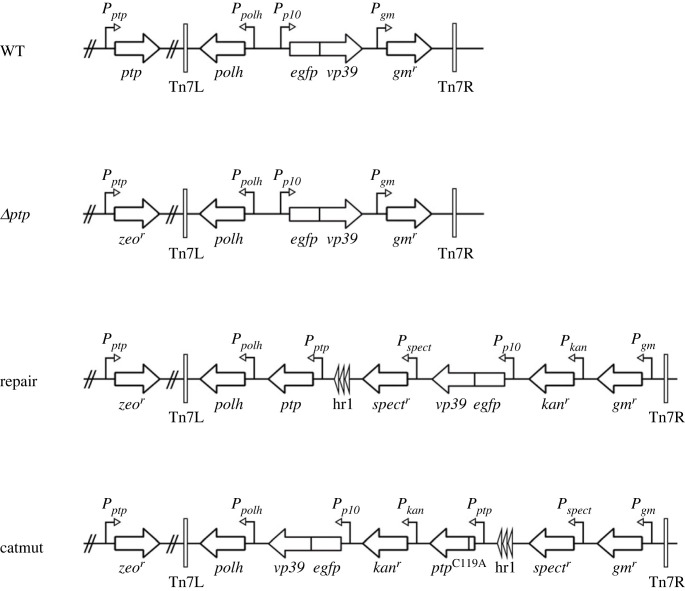
Overview of the modified parts of the recombinant AcMNPV viruses used in this study, including AcMNPV WT-eGFP (WT), AcMNPV Δ*ptp*-eGFP (Δ*ptp*) where *ptp* was replaced by a Zeocin resistance gene (zeo^r^), AcMNPV with *ptp* inserted back into the genome after deletion (repair) and AcMNPV encoding for a catalytic inactive PTP (catmut with the Cys-119 residue replaced with an alanine, C119A)). For all viruses, the polyhedrin (*polh*) gene and the fused *egfp-vp39* genes were inserted between the left and right insertion sites (Tn7L and Tn7R) present in the bacmids. For AcMNPV repair and AcMNPV catmut, the *ptp* gene was inserted with the upstream hr1 repeat region. Antibiotic resistance genes inserted during the different cloning steps [44] include the gentamicin (gm^r^), spectinomycin (spect^r^) and kanamycin (kan^r^) resistance genes.

The AcMNPV-eGFP:VP39 (WT-eGFP) construct [41] was used as wild-type (WT) in this study. This construct originates from the WT AcMNPV E2 bacmid [42,43] and contains a fused open reading frame (ORF) for enhanced GFP (eGFP) followed by the coding sequence for the AcMNPV major capsid protein (VP39) under control of the *p10* promotor (figure 2). The polyhedrin (*polh*) ORF is present downstream of the *polh* promotor. The fusion of eGFP to the N-terminus of VP39 allows subsequent visualization of the viral nucleocapsids and was shown not to affect viral infectivity, at least in cell culture [41]. In this construct, the original non-fused *vp39* gene is also present, at its normal locus.

In a similar way, a recombinant bacmid carrying the *egfp-vp39* fusion ORF was created using as basis a bacmid from which the *ptp* gene was removed (figure 2). Here nucleotide positions 509–1080 in the AcMNPV E2 bacmid, spanning the complete 507 nucleotides of the *ptp* ORF, were replaced with a zeocin resistance marker gene [23,45]. The *polh*-*egfp*:*vp39* expression cassette was transposed from a pFastBacDual construct [41] into the AcMNPV Δ*ptp* bacmid to generate the recombinant bacmid AcMNPV Δ*ptp*-eGFP:VP39 bacmid (hereafter called AcMNPV Δ*ptp*-eGFP).

To ensure that a potential phenotype after infection with AcMNPV Δ*ptp*-eGFP was not an expression of any additional genomic mutations when deleting the *ptp ORF*, a repair bacmid in which the *ptp* ORF was placed back was created (AcMNPV *ptp* repair). In addition, a recombinant bacmid encoding a catalytically inactive PTP protein (AcMNPV catmut) was created, to test whether the enzymatic activity of PTP is required for entry into the CNS. The catmut has a mutation in the HC signature motif where the Cys-119 residue was replaced with an alanine (C119A) [23]. The *ptp* ORFs (repair and catmut) were accompanied by an upstream hr1 repeat motif, known to enhance the expression of downstream genes [23,46]. The two ‘repair' bacmids were made using the MultiBac Expression system [44] (Geneva Biotech, Switzerland), as it allows for more than two gene inserts into the *polh* locus of a single bacmid. Both constructs were made starting from the AcMNPV Δ*ptp* bacmid mentioned above [45]. First the *polh* ORF was amplified with primers 1 and 2 (electronic supplementary material, table S1) providing XbaI and PstI sites, respectively, for cloning into the pACEBac1 acceptor vector downstream of its inherent *polh* promoter. The egfp:vp39 fusion mentioned above was amplified with primers 3 and 4 providing XhoI and NcoI sites for cloning behind a *p10* promoter in the pIDK donor vector, while the *ptp* or the *ptp–catmut* ORFs and the upstream *ptp* promotor and hr1 repeat region (as in [23]) were amplified with primers 5 and 6 (electronic supplementary material, table S1) providing SpeI and NsiI sites, respectively, for cloning into pIDS donor vectors. All amplified fragments were first cloned into pJET 1.2 (Thermo Fischer) and verified by sequence analysis before recloning in the respective acceptor or donor vectors. The pACEbac1-*polh* acceptor vector was fused with the pDK1-egfp:vp39 donor vector by Cre-LoxP recombination [44]. The resulting new acceptor vector was subsequently fused with the pIDS-ptp/catmut donor vector, also via Cre-LoxP recombination. The final transfer vector was used to transform DH10β ΔTN7 cells [47] carrying the AcMNPV Δ*ptp* bacmid leading to transposition of the multigene expression cassette into the mini Tn7 acceptor site at the *polh* locus.

### 2.3. Amplification and purification of virus

An existing OB stock of AcMNPV WT-eGFP [41] was amplified by oral infection (as described in §2.4) of early third instar larvae. A fluorescent signal in larvae was visually verified at 3 days post transfection using an UV-lamp under the stereomicroscope (Leica; Wild M3Z and HBO lamp). Liquefied larvae were collected and OBs were isolated by filtration over cheese cloth [23]. To obtain a virus stock of AcMNPV Δ*ptp*-eGFP, Sf9 cells were transfected with the bacmid and subsequently OBs were harvested and fed to *S. exigua* larvae. To that aim, the recombinant bacmid AcMNPV Δ*ptp*-eGFP was mixed with ExpreS2-TR Transfection reagent (Expres2ion) and transfected into Sf9 cells cultured in Gibco Sf-900 II serum free medium with 50 µg ml^−1^ gentamycin. A fluorescent signal was visible after 3 days post transfection and at 7 days post transfection, OBs were harvested by centrifugation (5 min. at 3000 r.p.m.). The pellet, containing the OBs and cell debris, was stored at 4°C and later fed to third instar larvae by droplet feeding (as described in §2.4). At 6 dpi, OBs were isolated from liquefied larvae by filtration over cheese cloth as described before [23].

To obtain OB stocks of AcMNPV repair and AcMNPV catmut, Sf9 cells were transfected with the bacmids as described above, using Lipofectin Reagent (Invitrogen, Fisher Inv.). At 7 days post transfection, BVs were harvested by centrifugation (10 min at 4100 r.p.m.) and supernatant, containing the BVs, was stored at 4°C until intra-haemocoel injection into fourth instar larvae. At 4 dpi, liquefied larvae were collected and OBs were isolated by filtration over cheese cloth [23]. The concentration of OBs in all stocks was estimated through counting in a haemocytometer (Thoma cytometer).

### 2.4. Oral infectivity assay

Late first and late second instar *S. exigua* larvae were starved overnight at 25.5°C, to secure ingestion of control (mock) or viral solutions the subsequent morning when the larvae had moulted into early second or early third instars, respectively. The treatments used for infections consisted of a mock 10% (w/v) sucrose solution laced with Patent Blue (Fluka Chemie GmbH), or viral stocks made in the same sucrose/Patent Blue mix, where OBs of the four viral constructs were diluted to a 10^8^ OBs ml^−1^ concentration, known to kill approximately 90–100% of the larvae [23,41] (corresponding to a >LC_95_, SN Gasque, unpublished results). A maximum of 30 larvae were placed in a 9 cm diameter Petri dish. The mock and OB suspensions were vortexed and 200 µl was offered as a circle of droplets around the larvae. Larvae were left to feed for a maximum of 15 min. The individuals that had ingested the solution (showing a clear blue gut), were placed individually in the wells of a 12-wells plate, each well containing a 8 × 8 × 8 mm feed block of artificial diet. Plates were covered by a piece of parafilm, a folded paper towel and the plate lid. Larvae were kept at 27°C until dissections at 1–7 dpi. Prior to dissection, eGFP expression was checked under an UV-lamp seen through a stereomicroscope, to ensure proper infection. Only virus-infected larvae expressing eGFP were selected, except for samples taken at 1 dpi where larvae were picked randomly, since eGFP expression was not yet detectable at this timepoint.

### 2.5. Dissections

Larvae were immobilized by placement on ice. One cold-immobilized larva at the time was decapitated with a sharp sterile scalpel at the second leg pair. Thereby the remaining sample included the head capsule and the pro- and mesothorax. This sample was moved to freshly made 4% PFA (4% paraformaldehyde (Merck) in 0.1 M phosphate buffer, pH 7.2) fixative in a dissection dish and placed under a light microscope (Leica Wild M3Z). The CNS was dissected out with sharpened forceps (Dumont nr. 5), by first making a rupture on the ventral side of the larvae, removing excess tissue and carefully tearing the oesophagus to facilitate intact circumoesophagal connectives and thereby connected ganglia. Sometimes tearing the oesophagus led to loss of the frontal ganglia and/or no connection between brains and SOGs. Each dissected CNS was carefully moved into fixative in Eppendorf tubes placed on ice, by grasping onto non-essential structures (such as remains of the trachea or connectives after the final attached ganglion), until the desired sample size was reached. The general outline of the fixation protocol has been described earlier [48], and was optimized for *S. exigua*.

### 2.6. Visualization

#### 2.6.1. For the time course of infection and different ganglia

Dissections were done for 7 consecutive days (1 dpi to 7 dpi). After the overnight fixation step in 4% PFA and 3 subsequent washing steps with phosphate buffered saline (PBS, Oxoid, Dulbecco ‘A' tablets), the samples dissected (*n* = 173 larvae) on the first six consecutive sampling days were stored in 1% sodium acid (10% in MiliQ water) in PBS—to prevent fungus growth—until all the dissected CNSs were ready to be taken through the subsequent protocol steps simultaneously. For the 1–6 dpi samples, the washings continued at day 8 with 2 more washings (a total of six washings with PBS). The 7 dpi samples were fixed overnight and then washed six times (no sodium acid added). To increase permeability of the neural tissue, the samples were incubated in 0.5 mg ml^−1^ collagenase (Sigma Aldrich) in PBS for 1 h at room temperature (RT), to digest the neurolemma. This was followed by three washings with PBS-T (0.5% Triton-X) and incubated overnight at 4°C of ThermoFisher TO-PRO-3 1 : 500 to label nuclei, in a 10% normal goat serum in PBS-T (PBS-T-NGS). The subsequent day the counterstain was washed away over 2 h by three washings of PBS-T, and over 2 h of three washings with PBS. The samples were dehydrated 2 min each in a series of 50%, 70%, 90%, 96%, 100%, 100% and then cleared by 50% xylene/50% ethanol, 100% xylene and lastly mounted in DPX on microscope slides. Owing to the sample multiplicity (173 CNSs) the samples were stored in PBS until they were dehydrated, cleared in xylene and mounted in distyrene plasticiser xylene mixture (DPX, Merck) on one of 3 consecutive days.

#### 2.6.2. For the protein tyrosine phosphatase CNS entry

CNSs for this experiment were dissected in the manner previously mentioned at 4 dpi using freshly made 4% PFA fixative and moved to Eppendorf tubes placed on ice, until the desired sample size was reached and fixed at RT for 2 h. Six washing steps with PBS (three quickly and following three in total of 1 h) or one overnight step at 4°C were used to wash off the fixative. Then all samples were incubated simultaneously in 0.5 mg ml^−1^ collagenase (Sigma Aldrich) in PBS for 1 h at RT. Subsequently washing away the collagenase in PBS-T (4 × 10 min), followed by a counterstaining step at 4°C overnight of TO-PRO-3 1 : 500 to label nuclei in PBS-T-NGS. The following day the samples were rinsed using several changes of PBS-T in a duration of 2 h and subsequently washed in PBS four times during 2 h. The samples were dehydrated 2 min each in a series of 50%, 70%, 90%, 96%, 100%, 100% and then cleared by 50% xylene/50% ethanol, 100% xylene and lastly mounted in DPX on microscope slides.

### 2.7. Image acquisition and scoring of infection

#### 2.7.1. Confocal laser scanning microscopy and imaging

For image acquisition, a Zeiss LSM 510 confocal laser scanning microscope (CLSM version 3.2 sp2) and a Leica Stellaris 5 CLSM were used. The LSM 510 was equipped with a 458/488-nm argon laser was used to visualize Alexa Fluor 488, and a 633-nm HeNe laser that was used to visualize TO-PRO-3. Scans were made ganglia per ganglia (or when fitting into the frame 2 at once) with an oil immersion objective; Plan-Neofluar 25x N.A. 0.8, in some cases the Plan-Neofluar 40x N.A. 1.3 was used and for details the Plan-Apochromat 63x N.A. 1.4 was used in very few cases. The resolution was kept at 2048 × 2048 pixels and 8 bits, with a voxel size of 0.078-0.078-1–0.225-0.225-2 µm for all images. The Stellaris 5 microscope possess a spectrally flexible white light laser with extended spectral output on the red and near-infrared spectrum (so to reach excitation from 405 up to 685). Samples scanned in similar fashion as at the LSM 510, ganglia per ganglia, now at the 40x N.A. 1.3 magnification comparable to the 25x N.A. 0.8 from the LSM 510 at eGFP at 488 nm and TO-PRO-3 633 nm by the LAS-X software package. In order to account for potential differences between the two confocal microscopes used in this study subgroups of samples were scanned on both microscopes and scored independently; however, no differences in scorings were found between scans at the two microscopes. Further analyses were conducted using the imaging software ImageJ 2.9.0 (Fiji Distribution) [49], the resulting figures were optimized for contrast by the levels command and the schematic overviews of the CNSs were made in Photoshop 24.4.1 (Adobe).

#### 2.7.2. Scoring of infection

A scoring system was developed to differentiate between different levels of infection. The judgement was made for each intact ganglia for each treatment and sample, and ranged from no infection to the most advanced infection level as follows: ‘no eGFP' (no eGFP expression detected at all), tracheal (‘tra’, eGFP expression detected only in the trachea, not in the CNS, and ganglial appendages), superficial (‘sup', eGFP expression detected in the outmost cell layers of the ganglia) and internal (‘int', eGFP expression could be detected in cells placed more internally than the outer cells of the ganglia) (see electronic supplementary material, dataset S3). For each timepoint, we recorded the most advanced level of infection at that moment.

### 2.8. Statistical analysis

The accumulated percentage of detected fluorescence in larvae of early second instar (eL2) and early third instar (eL3) AcMNPV-infected larvae (AcL2 and AcL3), did not follow a normal distribution. A simple survival analysis (Kaplan–Meier, log rank (Mantel–Cox) test) was used to test the two groups expression of fluorescence for similarity (significance level *α* = 0.05), to see whether the two groups could be considered as one and whether they could be grouped for the following analysis. Statistical tests were run and graphs were made in GraphPad Prism 9.5.1.

## 3. Results

### 3.1. Progression of virus infection within larvae and within the CNS

**Figure 3.**
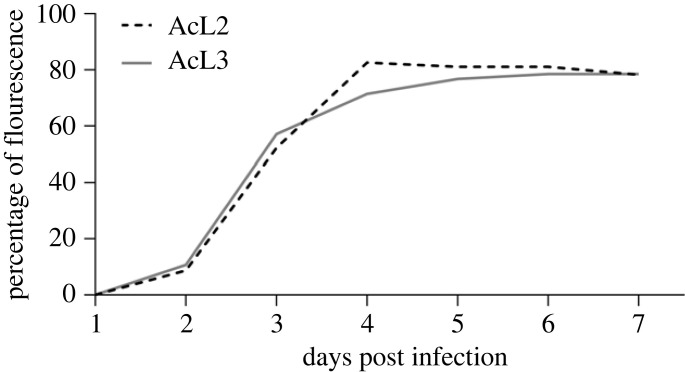
Accumulated percentage of *S. exigua* larvae infected with AcMNPV WT-eGFP (WT) showing external fluorescence. Larvae were examined daily from 1 to 7 days post infection (dpi) under a stereomicroscope with UV-lamp (Leica; Wild M3Z and HBO lamp), using 69 early second instar AcMNPV-infected (AcL2; dashed black line) and 56 early third instar AcMNPV-infected (AcL3; solid grey line) larvae. Percentages of fluorescence was the same during the course of infection for both treatments (Kaplan–Meier analysis, *p* = 0.69). For a few individuals the eGFP expression faded out after liquefaction and death. None of the (100) mock-infected individuals (ML2 and ML3) expressed eGFP at any dpi.

**Figure 7.**
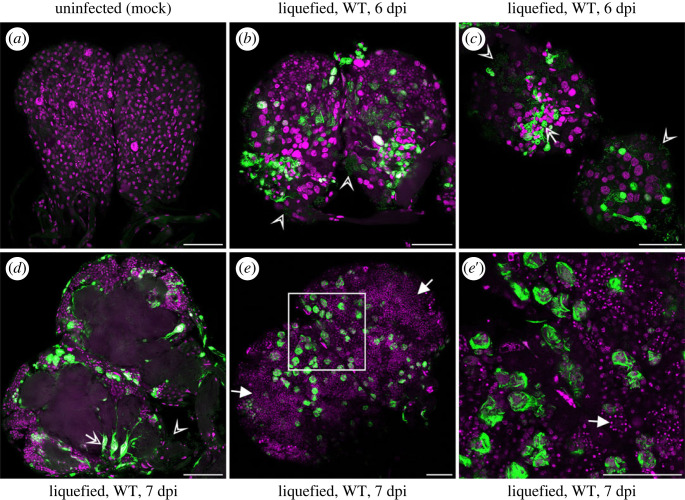
Confocal laser scanning microscope images of parts of the central nervous systems of liquefied *S. exigua* larvae (6 and 7 days post infection (dpi)) upon AcMNPV WT-eGFP infection. (*a*) Brain of a mock-infected larvae for comparison with (*b*) brain and (*c*) SOG and first thoracic ganglion (TG1) of 6 dpi liquefied larvae (both from larvae infected as early second instar) and (*d*,*e*,*e*′) brain of 7 dpi liquefied larvae (from larvae infected as early third instar). Open arrow heads in (*b*,*c*,*d*) indicate OBs released from cells post lysis, whereas the open arrow in (*c*,*d*) indicates OBs still enclosed in cells. Filled arrows in (*e*,*e*′) indicate lysed cells with fragmented nuclei (TO-PRO-3, magenta channel). (*e′*) is a magnification of the square area indicated in (*e*). All panels in the figure represent a stack on the surface of the ganglia, except for (*d*), where the internal infection is visualized from a stack in the midline of the brain. A white signal indicates an overlap and high intensity of the green (AcMNPV WT-eGFP) and magenta channel. Scale bars represent 50 µm.

Virus progression within larvae (*n* = 225) and within the CNS of individuals from the same group (*N* = 173, which per different ganglia resulted in 700 individual scorings of infection levels) was investigated for AcMNPV WT-eGFP-infected larvae, with mock-infected larvae as a control. In none of the mock-infected larvae an eGFP signal was detected, neither when checking larvae externally under an UV-lamp (*n* = 100), nor in the dissected CNS scanned at the CLSM (*n* = 68). At day 0 and 1 dpi no external eGFP expression could be observed for the AcMNPV-infected larvae by checking under a UV-lamp. This changed at 2 dpi when external eGFP expression was observed in up to 11% of the WT-eGFP-infected larvae, increasing to 52–57% at 3 dpi and with a peak at 4 dpi of 71–83% (figure 3; electronic supplementary material, dataset S1). The following 2 days (5–6 dpi) the percentage was 77–81%, and at the last recording day (at 7 dpi) this percentage of external eGFP expression was maintained or slightly decreased. On day 6 and 7, some of the larvae started dying and liquefying (figure 7), and eGFP expression ceased (figure 3). The percentage of larvae observed expressing external fluorescence did not significantly differ between larvae infected as eL2 or eL3 (figure 3; *p* = 0.69; electronic supplementary material, dataset S2), and therefore the data were pooled for the subsequent analyses.

**Figure 4.**
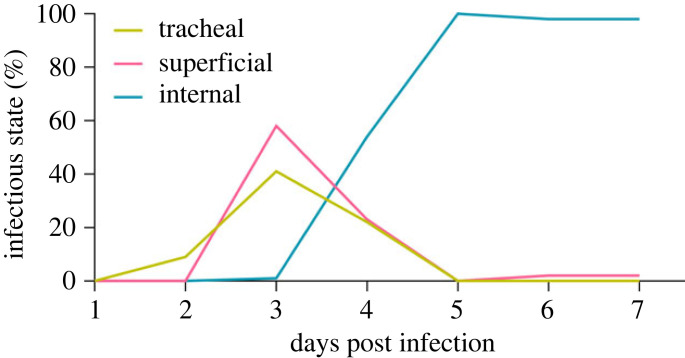
Percentage of AcMNPV WT-eGFP-infected larvae (infected as second and third instars) expressing eGFP only in trachea (‘tracheal'; yellow line), or also superficially in the ganglia (‘superficial'; pink line), or also internally in ganglia (‘internal’; blue-green line) for all the analysed ganglia (brains to first abdominal ganglia) at different days post infection. This figure visualizes input data from 413 individual observations of infection levels in different ganglia (*n* = 32 for 1 dpi, 53 for 2 dpi, 69 for 3 dpi, 90 for 4 dpi, 77 for 5 dpi, 51 on 6 dpi and 41 for 7 dpi) from a total of 105 AcMNPV WT-eGFP-infected larvae, and does not include the data from the mock-infected larvae (*n* = 68) as none of the scans of 286 ganglia showed any eGFP expression.

**Figure 5.**
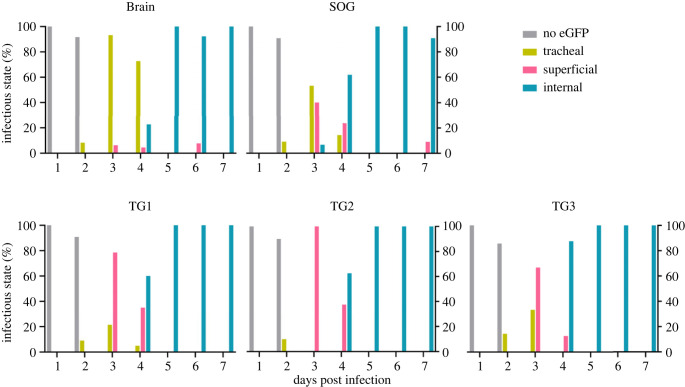
Percentage of AcMNPV WT-eGFP-infected larvae (infected as second and third instars) expressing no eGFP (‘No eGFP'; grey bars), eGFP only in trachea (‘tracheal'; yellow bars), or also superficially in the ganglia (‘superficial'; pink bars), or also internally in ganglia (‘internal'; blue-green bars) for the brain, SOG and first to third thoracic ganglia (TG1, TG2 and TG3) at different days post infection. The data represent the most advanced level of infection observed (see §2.7 scoring of infection). This figure shows data from 402 individual scorings of ganglia (data on AG1 are not represented in this figure).

**Figure 6.**
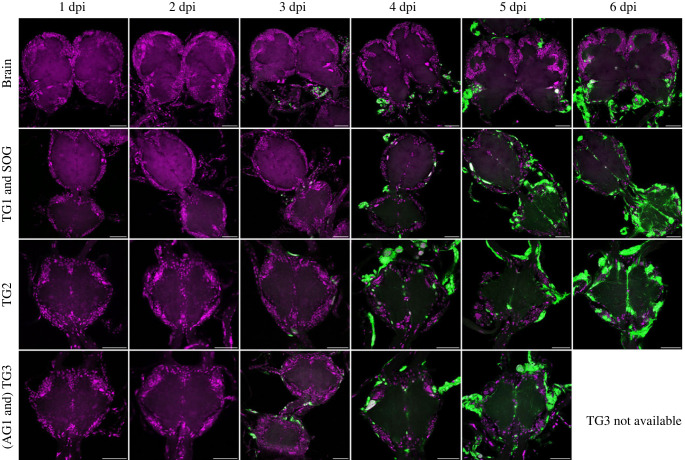
Confocal laser scanning microscope images of AcMNPV WT infection in the brain, SOG, first, second and third thoracic ganglia (TG1–TG3), and the first abdominal ganglia (AG1; for 3 days post infection (dpi) only) at 1–6 days dpi. All images represented here are from *S. exigua* larvae infected as early third instars with a 10^8^ OBs/ml concentration. Magenta channel for the TO-PRO-3 nuclei/dsDNA staining, green channel for AcMNPV WT-eGFP. White signal indicates an overlap and high intensity of the green and magenta channel. Scale bars represent 50 µm.

For the WT-eGFP-infected larvae, viral infection of the trachea surrounding the CNS became visible from 2 dpi, and at 3 dpi viral infections were observed in the trachea for all larvae and stayed persistent through the observation days (figures 4–6). From here onwards (3 dpi) the first viral infections of the cell bodies in the outer layers of the different ganglia could be observed (termed as superficial infection; figures 4–6). Internal infection (in the inner layers of the ganglia) could only be observed in one individual (out of 15) at 3 dpi, in the SOG (figure 5; electronic supplementary material, dataset S3). From 4 dpi, we started to observe more infections of the internal layers of cell bodies in the different ganglia (54.4%) and from 5 dpi onwards the infection intensified as nearly all ganglia showed infection in the internal neural layers (figures 4–6; electronic supplementary material, dataset S3).

**Figure 8.**
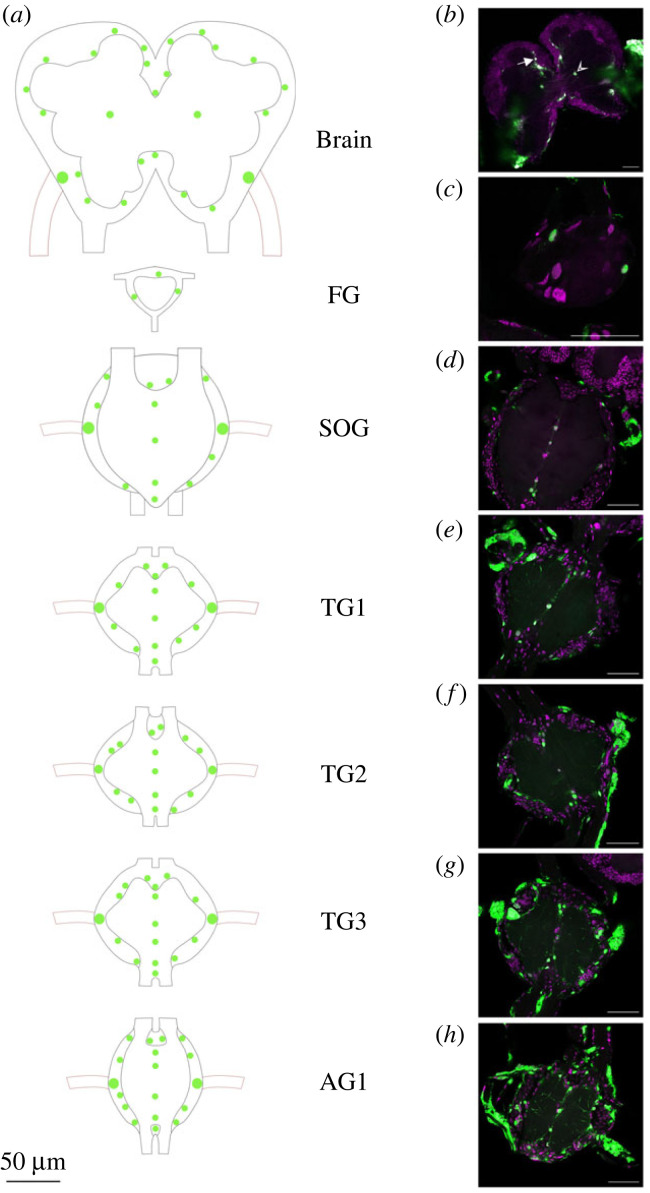
(*a*) Schematic overview of AcMNPV WT-eGFP localization in the CNS (from brain to first abdominal ganglia (AG1)) *of S. exigua*, with (*b*–*h*) representative images of confocal laser scanning microscope (CLSM) imaging for each of the analysed ganglia. Based on CLSM images, the major localizations of AcMNPV WT-eGFP were visualized with green dots in (*a*), and trachea are visualized as appendages to the ganglia with red-brown lines (viral infections in the trachea are not represented in the schematic overview). (*b*) Brain with filled arrow indicating infected cell bodies surrounding the calyx and open arrow head indicating infected cell body in the centre of the superior neuropil; (*c*) frontal ganglion (FG); (*d*) SOG; (*e*) first thoracic ganglion (TG1); (*f*) second thoracic ganglion (TG2); (*g*) third thoracic ganglion (TG3); (*h*) first abdominal ganglion. (*b–h*) are from 3 to 5 dpi; representative images were picked to illustrate the overall trends of viral progression and localization. Magenta signal for the TO-PRO-3 nuclei/dsDNA staining, green channel for AcMNPV WT-eGFP. Scale bars represent 50 µm.

Over the course of infection a pattern became visible, with certain areas and ganglia being infected earlier than others. Hence, the expression of eGFP was first detectable in the trachea surrounding the CNS, then in the outer cell layers and lastly in the internal layers of the ganglia (figures 4–6). Alongside this infection by layers, the infection also progressed with respect to the ganglia (figure 5). The infections in the superficial cell layers and later the more internal layers were first detected in the abdominal ganglia and the three thoracic ganglia, then in the SOG and lastly in the brains along with the frontal ganglia in some occasions (figure 8).

#### 3.1.1. Central nervous systems from liquefied larvae

Of the few liquefied larvae where it was still possible to dissect out complete ganglia (*n* = 13 larvae, *n* = 46 individually scored ganglia), the lysis of cells could be observed by the decreased and fragmented area for the TO-PRO-3 signal (staining dsDNA) and by OBs observed within and outside of cells (eGFP signal) (figure 7). Some cells are found highly enlarged, just prior to lysis (figure 7*ee*′). The ‘banded' WT-eGFP perinuclear structure superficially located on the ganglia (figure 7*b*,*c*,*e* and *e*′) had a high resemblance with previously observed patterns in Sf9 cells infected with the same AcMNPV WT-eGFP virus as used in our study (AcMNPV-eGFP:VP39; [41]) and in AcMNPV-infected TN-368 cells, where a P10 cage-like structure was seen [50] (electronic supplementary material, figure S1). The *p10-*gene (ac137) has been described as being transcribed very late in baculovirus infection, and the P10 (10 kDA) cage functions in breaking the nucleus and thereby aids in the OB release [15,51]. We assume that the visualization of the banded structure is due to precipitation of VP39-eGFP with P10 when the VP39 is over-expressed, e.g. in the example of liquefied larvae (figure 7).

### 3.2. Specificity of viral localization

The observed patterns of viral localization throughout the analysed ganglia were similar for all samples, with certain cell bodies being infected more frequently than others (figure 8). In the brain, the cell body in the centre of the superior neuropil [52] (figure 8*a,b* open arrow head) and the cell bodies near the trachea (figure 8*a,b*) were frequently infected. When the brain was more heavily infected, we observed infection of the cell bodies surrounding the superior neuropil, e.g. those surrounding the calyx (figure 8*a,b* filled arrow). For the SOG (figure 8*a,d*) and the subsequent thoracic ganglia and first abdominal ganglion (figure 8*a,e–h*), the most frequent pattern was the infection of cell bodies that were localized in a midline of either of the ganglia, or in close proximity to the trachea, connectives and nerves (figure 8*a,e–h*). When the SOG and the following ganglia were more heavily infected, the infection could furthermore be localized to cell bodies surrounding the neuropil, similar to what was seen for the brain. In heavily infected brains, the axons could sometimes be seen, but the axon signal was more clearly visible in the SOG or in the first, second, third thoracic ganglia or first abdominal ganglia (see 4–6 dpi in figures 6 and 8*e–h*). In some occasions superior or internal infections of the frontal ganglia could also be detected (figure 8*a,c*).

### 3.3. The role of protein tyrosine phosphatase in CNS entry

**Figure 9.**
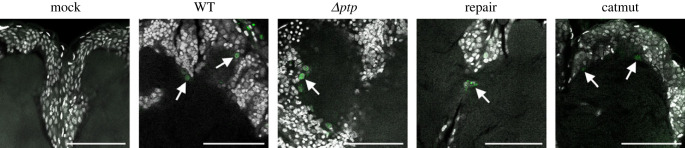
Confocal laser scanning microscope images of the same brain area, showing the calices (all) and/or midline (mock, repair, catmut) of brains dissected at 4 days post infection from larvae infected as early third instar with a mock solution (Mock), or with one of the recombinant AcMNPV viruses expressing eGFP (AcMNPV WT-eGFP (WT), AcMNPV Δ*ptp*-eGFP (Δ*ptp*) lacking *ptp*, AcMNPV with *ptp* repaired back after deletion (repair) and AcMNPV encoding for a catalytic inactive PTP (catmut). White signal for TO-PRO-3 nuclei/dsDNA staining, green channel for eGFP signal from the recombinant viruses described. Scale bars represent 50 µm.

Since the enzymatic active form of PTP is essential for the expression of hyperactivity in the AcMNPV-*S. exigua* system [23], we tested whether viral entry into the CNS, the area for movement initiation, is dependent on PTP. The earliest and the clearest infections patterns could be detected superficially and internally from 4 dpi in AcMNPV WT-eGFP-infected larvae (figure 6; see 3.1). We set out to test whether the other AcMNPV-variants could also enter the CNS, and if so, whether there were differences in localization (described for WT-infected larvae in 3.2). In the CNSs dissected out from larvae orally infected with any of the AcMNPV-variants used in this study, eGFP expression was detectable from 4 dpi (figure 9). Neither the enzymatic function nor the structural properties of AcMNPV-PTP seemed essential for CNS entry in *S. exigua*, as all recombinant viruses—including the virus without *ptp*—could enter the CNS. Furthermore, no differences were seen for either the superficial or the internal infections of the localization in the CNS for the different recombinant viruses in this set-up.

## 4. Discussion

Baculoviral modification of caterpillar behaviour is a clear example of behavioural alteration by parasites. As the CNS can serve as starting point in the initiation of a range of behaviours, we followed a neuroparasitological approach [38] to investigate if, when and where AcMNPV infects the CNS of *S. exigua* caterpillars. Furthermore, we addressed the possible role of viral-PTP in the entry of AcMNPV into the CNS. Since the various ganglia process the reception and send signals involved in different actions and behaviours, we investigated each ganglion (up to the first abdominal ganglion) separately. Our findings reveal that AcMNPV enters the CNS from 3 dpi onwards, most likely via the trachea. The virus signal in the CNS is first visible in the outer cell layers (e.g. in the cell bodies near the connectives). Subsequently from 3 dpi onwards the infection spreads to the more internal cells. Within the CNS, the first to third thoracic ganglia and the first abdominal ganglion are infected first, followed by the SOG, then the brain and the frontal ganglion. For the internal infections, certain cell bodies are infected more frequently than others. In the SOG, thoracic ganglia and abdominal ganglia, the most commonly infected internally layered cell bodies are found in the midlines of the neuropil and in close proximity to the trachea, connectives and nerves. This is the same for the brain, where in addition infected cell bodies are found in close periphery to the superior neuropil or in the centre of the superior neuropil. Neither the enzymatic function, nor the structural properties of AcMNPV-PTP are needed as differences were neither seen in the superficial nor the internal infections by these differently *ptp*-expressing viruses.

### 4.1. Progression of infection

From 3 dpi onwards, we observed superficial infection (followed by internal infection) of the CNS. While in our virus constructs, GFP fused to VP39 is expressed under the *p10* promoter, which is a very late promoter (as reviewed by van Oers [53]), we still think that GFP observations at 3 dpi reflect virus entry into the CNS. Previous studies showed that *p10* transcripts start to be produced from 18 hpi onwards [54] and that fused and unfused VP39 (which was not removed from the viral genomes and is expressed under its own promoter) are both incorporated into the BVs, as has been shown before [41]. This indicates that fluorescing nucleocapsids (and BVs) will be visible from about 18 hpi. In our earlier work, GFP was incorporated in the nucleocapsids, and since the timing of nucleocapsid and BV production has not changed [41], our observations are likely to reflect AcMNPV entry into the host CNS.

In an earlier study, infections of neural glial cells have been described from 2 to 3 dpi in the same virus–host system [35]. In our experimental set-up, the first signs of viral infection (visible through the cuticle due to eGFP expression) were detectable from 2 dpi onwards and at the same day we started to see infections in the trachea. This corroborates with results from other studies, such as for Helicoverpa armigera NPV-infected cotton bollworms, where the first viral infections were detected by GFP-expression at 2 dpi in epithelial cells and trachea [36]. After the initial infection of the midgut epithelial cells, neighbouring cells as well as the tracheal cells along with haemocytes are the first cells to get infected by BVs in the secondary phase of baculoviral infection [13,16,55,56]. The trachea have also been shown to be the major route of AcMNPV transmission through *Trichoplusia ni* caterpillars, and as the trachea branch into the CNS by tracheoles this route seems viable [37,55,57,58].

Our study provides data on tracheal infection prior to superficial and internal infections of the different ganglia. The eGFP signal detected from the trachea was a lot stronger than from any of the ganglia and stayed persistent throughout. As the trachea are connected to the ganglia and branch within, the tracheal cells border the two types of glia cells (perineurial and subperineurial) and the neural lamella which comprise the blood–brain barrier surrounding the CNS [59–61]. With our experiment, we cannot out rule that other cell types (e.g. haemocytes) are also in direct contact with the cells that comprise the blood–brain barrier and that these cells might also play a role in the infection of the CNS. Owing to the BVs mode of infecting neighbouring cells, the baculoviral cell to cell infection could provide an alternative route compared to the route other pathogens must follow to surpass the blood–brain barrier to infect the CNS, such as the Trojan horse strategy (phagocytized by macrophages) [62–64]. The earlier described banded pattern displayed by VP39-eGFP (figure 7*e*,*e*′; electronic supplementary material, figure S1) in the enlarged cells on the surface of the liquefied brains was also observed in the trachea. The fact that we also found indications for VP39 precipitation on baculoviral P10 structures in tracheal cells (*data not shown)* when VP39-eGFP is over-expressed from the highly active *polh* promoter (similar to what is reported in electronic supplementary material, figure S1) shows that infection proceeds into the very late phase also in trachea. This finding further supports the route of transmission via trachea for surpassing the brain barrier. The fact that our results always indicate a high amount of viral structural proteins (shown by VP39-eGFP) in the trachea, is coherent with previous studies. Increased relative expression of the viral structural gene *gp64* was, for instance, seen in the trachea at 3 and 4 dpi compared to the fatbody at 4 dpi of BmNPV-infected *B. mori* caterpillars [34]. Owing to the fact that we see tracheal infection prior to CNS-infection and the strong continuous infection of the trachea compared to the CNS, we hypothesize that the trachea are the route for viral infection of the CNS.

The more caudally (towards the rear end) located ganglia are closer located to the alkaline zone of the midgut (where the OBs dissolve), than the more anterior ganglia (such as the brain and SOG) [65]. We found a link between increase in infection level connected to increasing days after infection and the order of infection of the different ganglia in the CNS. Generally, the further caudally the ganglia were located, the earlier more advanced infection levels were observed. This positive link between more advanced infection levels of the more caudally located ganglia and the role of thoracic and abdominal ganglia in the initiation of movement [66], suggests that the infection of these ganglia is also related to the induced changes in behaviour.

Cells involved with movement can be found on the external/superficial part of the ganglia [67], where we also observed virus infections. In many cases, we observed an enlargement (compared to neighbouring cells and mock controls) of the infected neural cells adjacent to the trachea. Given the size of the cell bodies these cells could be glia cells, but the enlargement might very well be due to the viral infection and then they could also represent neurons. Interestingly, the infection does not simply spread from initially infected cell to directly neighbouring cells, since we do not see every cell body being infected from the surface of a ganglion to the most internal parts (such as the infections of the midlines of the SOG, first–third thoracic ganglia and the first abdominal ganglion). Also, the infection of a single cell (to a few) in the centre of the superior neuropil of the brain is remarkable. We currently do not know which cell type is infected in the latter, but tracheal cell bodies have been described around the same location in *Drosophila* [68,69]. If these cell bodies are indeed tracheal cell bodies in *S. exigua,* this would further support our theory of viral CNS-infection through the trachea as these cells had been infected in nearly all cases. Furthermore, eGFP signal was detected in some axons indicating that the infection might progress through the axons when having entered the CNS, e.g. to the internally located and centralized cell bodies in the (superior) neuropil in the different ganglia. Axonal invasion and transport via the axons have previously been described for the much smaller RNA virus, Rice yellow stunt virus (RYSV; family *Rhabdoviridae*) in the CNS of the green rice leafhopper (*Nephotettix cincticeps*) [70].

### 4.2. Specificity of viral localization in the CNS

Viral infection of the CNS does not follow a random pattern, in contrast, we found that the virus localizes at specific regions in the ganglia (figure 8). For most of the infected cells in the CNS, the function of these cells is currently unknown and it is also uncertain whether these cells are glial cells or neurons. Further research is needed to see whether AcMNPV specifically infects cells with a particular function, and whether the presence of AcMNPV at these locations has an influence on the induction of hyperactivity. As mentioned above, AcMNPV often infects cells in the midline of the ganglia of the SOG and the ganglia caudally placed, and these are potentially midline glia (MG) cells [71,72]. The infected cells in the centre of the superior neuropil in the brain are in close proximity to different parts of the mushroom body (the central processing unit involved in associative learning and memory) and the central complex (involved in vision-mediated behaviours and spatial information with locomotor control) [73–75], it remains to be studied whether AcMNPV specifically infects those cells to induce hyperactivity.

The viral infection of cell bodies in the rim of the superior neuropil can be further split down to the rim of the calyx (part of the mushroom body), the rim of the optic lobe (perception of signals from the eyes) and the rim of the antennal lobe (perception of signals from the antennae), which indicates that a whole range of different functions could be at play for the viral locations observed in the inner layers of the ganglia. The cells that we detected in the heavier infected samples that are located at the rim surrounding the superior neuropil are likely to be neurons [66] and with the detection of axonal infections we have strong indications of neuronal infections by AcMNPV in *S. exigua*.

Interestingly, the pattern of the above-mentioned localization of AcMNPV-infected cells in the CNS of *S. exigua* seems to overlap with the expression of ecdysone receptors (EcRs) shown for *Drosophila melanogaster* and *Manduca sexta* [68]. Ecdysone receptors A and B1 (EcR-A and EcR-B1) are located in the MG, the glia of the optic lobe, the perineuropilar glia and in neurons of the mushroom body and of the optic lobe of *D. melanogaster* and *M. sexta* [68]. The insect steroid ecdysone I is required during moulting and metamorphosis, and a peak in relative levels of *D. melanogaster* larval EcR-B1 was detected during the wandering stage [68,71]. The active state of E, 20-hydroxyecdysone (20E), can be inactivated by ecdysteroid UDP-glucosyl transferase (EGT) (which is present in nearly all baculoviruses) which thereby stalls the normal moulting process of some caterpillars infected by baculoviruses. Furthermore, EGT might play a role in virus-induced tree-top disease and hyperactivity [11,20,76–78] (reviewed by Gasque *et al*. [12]) and therefore it will be highly interesting to further study the link between AcMNPV-infected cells and the expression of EcRs.

### 4.3. PTP-induced hyperactivity by baculoviruses

Protein tyrosine phosphatase is a factor needed for initiating hyperactivity in BmNPV-infected *B. mori* caterpillars [22]. The same is the case for AcMNPV-induced hyperactivity expressed by *S. exigua* at 3 dpi [23]. Despite the presented similarities, a major difference between the two systems is seen in the exact role of PTP. For BmNPV-infected *B. mori* solely the structural properties of PTP are needed, whereas for AcMNPV-infected *S. exigua* caterpillars the hyperactivity will only be expressed if AcMNPV also encodes for the enzymatic function of PTP [23,34]. As we have shown in this study, all the eGFP-expressing AcMNPV recombinant viruses could enter the CNS of *S. exigua* including AcMNPV with a deleted *ptp* gene and the virus with a catalytically impaired PTP. We can therefore conclude that for AcMNPV-infected *S. exigua*, PTP is non-essential for CNS entry, and our data therefore does not support the suggested hypothesis that baculoviral PTP aids in CNS entry of caterpillars [34]. Both AcMNPV and BmNPV belong to group I NPVs wherein the *ptp* gene is conserved [23]. Since group II NPVs also induce hyperactivity [18], but do not encode PTP, it is certain that other mechanisms than solely PTP can induce hyperactivity in the baculoviral-lepidopteran system.

In conclusion, we show the progression of a baculovirus infection in the CNS most likely by the entry via trachea, of initially the more caudally located ganglia placed closer to the alkaline zone of the midgut, followed by the SOG and the brain. Furthermore, we have shown infections of glia cells and neurons, in external and internal layers of all the ganglia, along with AcMNPV's independence of PTP for the entry to the CNS of *S. exigua*. Further research is needed to identify possible interactors of PTP and to disentangle the pathway downstream, but also the search for other possible non-PTP interacting mechanisms that aid to express hyperactivity is needed. Our results demonstrate, that although PTP is not needed for AcMNPV to enter the CNS, the location pattern still suggest a role in manipulating the host nervous system, adding novel insights into the mechanism underlying this intricate host–parasite interaction.

## Data Availability

The data are provided in electronic supplementary material [79].
